# A Window of Opportunity to Control the Bacterial Pathogen *Pseudomonas aeruginosa* Combining Antibiotics and Phages

**DOI:** 10.1371/journal.pone.0106628

**Published:** 2014-09-26

**Authors:** Clara Torres-Barceló, Flor I. Arias-Sánchez, Marie Vasse, Johan Ramsayer, Oliver Kaltz, Michael E. Hochberg

**Affiliations:** 1 Institut des Sciences de l'Evolution, CNRS-Université Montpellier 2, Montpellier, France; 2 Santa Fe Institute, Santa Fe, New Mexico, United States of America; 3 Wissenshaftskolleg zu Berlin, Berlin, Germany; Charité-University Medicine Berlin, Germany

## Abstract

The evolution of antibiotic resistance in bacteria is a global concern and the use of bacteriophages alone or in combined therapies is attracting increasing attention as an alternative. Evolutionary theory predicts that the probability of bacterial resistance to both phages and antibiotics will be lower than to either separately, due for example to fitness costs or to trade-offs between phage resistance mechanisms and bacterial growth. In this study, we assess the population impacts of either individual or combined treatments of a bacteriophage and streptomycin on the nosocomial pathogen *Pseudomonas aeruginosa*. We show that combining phage and antibiotics substantially increases bacterial control compared to either separately, and that there is a specific time delay in antibiotic introduction independent of antibiotic dose, that minimizes both bacterial density and resistance to either antibiotics or phage. These results have implications for optimal combined therapeutic approaches.

## Introduction

Antibiotic resistant bacteria are a widespread problem that threatens human health. Due to the rapid adaptation of bacteria to old and new antibiotics there is an urgent need to develop alternative treatments [1] [2] [3]. Phage therapy, the use of parasitic viruses as antibacterial agents is attracting renewed attention due to their host specificity, innocuity for treated patients, and potential for evolution to outpace bacterial resistance [4]. Despite considerable research on single or combined therapies involving phage [5] [6], the underlying evolutionary processes remain poorly understood.

Evolutionary theory predicts that combined therapies can be more effective than a single component agent for preventing or limiting the evolution of antibiotic resistance [7], and this approach has gained attention in the control of pathogenic microbes [8] [9]. Specifically, adaptive trade-offs can emerge due to fitness costs associated with resistance to more than one antimicrobial agent, as shown in the evolution of resistance to multiple antibiotics [10]. Despite their potential, combined antimicrobial therapies are subject to the evolution of resistance due to convergent mechanisms of resistance if they target similar pathways, and the specific combination will determine the speed of resistance evolution [11]. Synergistic drug combinations, where joint antimicrobial effectiveness is greater than the individual effects, are more efficient and can be employed at lower doses, although selection for resistance can be substantial [11] [12]. Antagonistic drugs have a combined effect that is lower than predicted, and although they generally slow the evolution of resistance are rarely used in a clinical context [11] [12].

The actual implementation of antibiotic therapies also has important implications for the development of resistance [13] [14]. For instance, antibiotic dose can have an important effect on the evolution of resistance, but the mechanisms involved differ between low and high doses. In general, lower doses select for low cost resistance mutations that can be crucial to the stepwise acquisition of higher dose resistance, and higher doses impose stronger selection for resistant alleles [15] [16]. Another factor influencing the short and long-term efficiency of combined therapies is the timing of application, especially for antibiotics and phage, where phage replication and antibiotic effect are both density-dependent [17]. Phage population dynamics will be determined by the number of hosts in which they can replicate, with consequences for the amplification of phage densities and the therapeutic effectiveness [18]. If phages are administered at low bacterial densities or bacteria non-amenable physiologically, then the increase in phage densities will be lower and recurrent application of phages may be necessary [18].

We challenged the opportunistic pathogenic bacterium *Pseudomonas aeruginosa* PAO1 with a lytic bacteriophage and the antibiotic streptomycin, with the aim of uncovering the effects of independent and combined treatments. This nosocomial pathogen species represents a particular danger to cystic fibrosis patients, and is known to readily evolve antibiotic resistance [19]. The antibiotic streptomycin has been shown to act synergistically when used with other chemical antimicrobials and is commonly used to treat *P. aeruginosa* infections [20] [21]. By studying *in vitro* bacterial density dynamics, we show that phages and streptomycin have a synergistic negative effect against bacteria. We also find a specific window of opportunity in the addition time of the antibiotic, enhancing the suppression of populations already treated with phage. Antibiotic dose did not significantly affect bacterial density, contrary to conventional clinical practice of using high antibiotic doses [16]. Finally, we find no evidence that the synergistic effect of the combined treatments is driven by genetic trade-offs between resistances to the phage and to the antibiotic. A more likely explanation is a demographic feedback produced by phage addition, limiting the capacity of the bacteria to resist antibiotic exposure. Our study provides an evolutionary basis for the optimization of combined treatments.

## Materials and Methods

### Bacterium, phage and media

We used the bacterium *Pseudomonas aeruginosa* PAO1 and the phage LUZ7, from the *Podoviridae* family [22]. The experiment was carried out in 24-well plates, with bacteria growing in King's B (KB) medium at 37°C without agitation. M9 medium was used for dilutions. The antibiotic streptomycin (Sigma-Aldrich) was added to liquid medium at either 100 or 240 µg/mL, known to represent sub-lethal and MIC concentrations for PAO1, respectively [10]. The phage stock was prepared as described in [23]. Briefly, 10% vol/vol chloroform was added to phage-containing bacterial cultures, vortexed and centrifuged. Phage-containing supernatants were carefully recovered and stored at 4°C. This LUZ7 stock (10^7^ PFU/mL) was used as the ancestral phages for all the experiments.

### Experimental design

**Figure 1:**
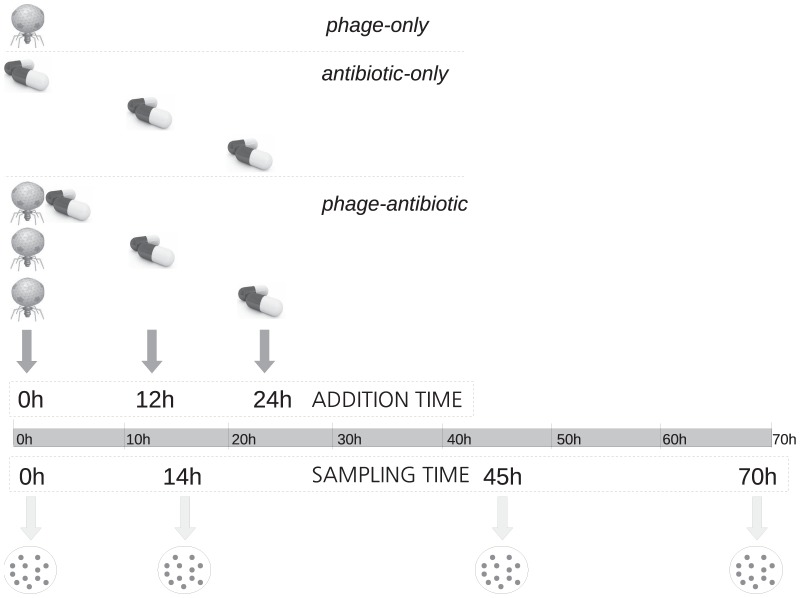
Overview of the experimental design. Exponentially growing bacteria were treated with (i) only phage (at 0 h), (ii) only antibiotic (1 dose at 0 h, 12 h, or 24 h) or (iii) first phage and then antibiotic (1 dose at 0 h, 12 h, or 24 h). Replicate populations of the bacteria were sampled at 0 h, 14 h, 45 h and 70 h, for density and resistance measurements. All antibiotic treatments were repeated for two streptomycin doses (100 and 240 µg/mL).

Six hours prior to the start of treatments, the 120 bacterial replicate populations were initiated from a *P. aeruginosa* PAO1 overnight culture, by adding 15 µL of culture to 1.5 mL of KB in 24-well plates. Phages were added (10^5^ LUZ7 phages/mL) after 6 h (T_0_), when bacterial populations were growing exponentially and therefore vulnerable to phage attack. We used a concentration of phages high enough to affect the bacterial population dramatically (decreasing density by 6 orders of magnitude), but without producing complete extinction. We established single treatments, with only phage or only antibiotic added, as well as combined phage-antibiotic treatments (Figure 1), named single-phage, single-strep and phage-strep, respectively. The antibiotic was added at one of three time points: simultaneously with the phage (+0 h), with a delay of +12 h, or with a delay of +24 h. Two antibiotic doses were tested: 100 or 240 µg/mL. For each treatment we established 9 replicate populations, 108 total: 2 phage (yes/no) x 2 antibiotic doses x 3 addition times x 9 replicates. Six control replicate lines were established for the single-phage treatment and for untreated control lines.

### Density measurements and resistance assays

Bacterial density was measured at different time points (T_0_, T_14_, T_45_, T_70_ = 0, 14, 45, and 70 hours post phage inoculation), by counting the number of growing colonies (colony-forming units, CFU) from samples plated on KB agar at appropriate dilutions.

At the end of the experiment (T_70_), we assessed the surviving populations' resistance to streptomycin. 1 µL of the final populations were inoculated on to 250 µL of fresh KB containing streptomycin at different concentrations (12, 25, 50, 100, 200, 400 or 800 µg/mL). After 24 h, bacterial density was measured by means of optical density (OD) at 600 nm (Fluostar, BMG LABTECH). Resistance was taken as the Minimum Inhibitory Concentration (MIC), defined as the streptomycin concentration at which no bacterial growth was detected. For populations even resisting the highest concentration (800 µg/mL) the MIC was arbitrarily set to 1600 µg/mL.

To measure phage resistance, 1 µL of final bacteria was added to 250 µL of media containing ancestral phage (*c* 10^5^ phages) and OD recorded after 24 h. Phage resistance was taken as a quantitative trait, calculated as the difference in OD obtained with and without phage added. The same assay was performed with evolved phage. To this end, the 9 replicates from the last time point of a given treatment were pooled and evolved phages extracted as described above. Thus, bacteria were confronted with a mix of phages from their own treatment. Bacteria from treatments without phage (single-strep, control, ancestral bacteria) were confronted with evolved phage from the +0 h phage addition time treatment with 100 µg/mL of streptomycin. All OD values were corrected for absorbance of blank wells; replicates for which positive control wells without phage showed zero growth were not used for analysis.

### Statistical analysis

Using the JMP statistical package [24], we employed General Linear Model (GLM) techniques to analyze variation in bacterial density (CFU/mL, log_10_-transformed), antibiotic resistance (MIC, square-root-transformed) and phage resistance (OD difference between bacteria challenged with phage and not). In the main analyses, we tested fully factorial models, containing phage treatment (yes/no), antibiotic dose as explanatory factors and antibiotic addition time as a covariate. To test for non-linear effects of addition time, we also fitted its second-order polynomial term (addition time^2^). Minimal adequate models were established through backward elimination of non-significant terms in the model. Where appropriate, analyses were carried out separately for single and combined treatments; additional tests compared evolved and ancestral bacteria.

To calculate expected final densities (70 h) in combined phage-antibiotic treatments, we paired single-phage with single-strep replicates. For both replicates in a pair, we calculated the reduction in bacterial density relative to the untreated controls (difference in CFU/mL). We then added together the two single density reductions to obtain the expected density in a hypothetical combined phage-antibiotic treatment. Specifically, for each combination of antibiotic dose and addition time, 36 of the possible 81 (9×9 replicates from single treatments) pairs were arbitrarily chosen and the density difference calculated relative to each of the two untreated control lines. This gave a total of 72 expected values that were to be compared with the corresponding observed values in the true combined phage-antibiotic treatment.

## Results

### 1. Bacterial density

**Figure 2:**
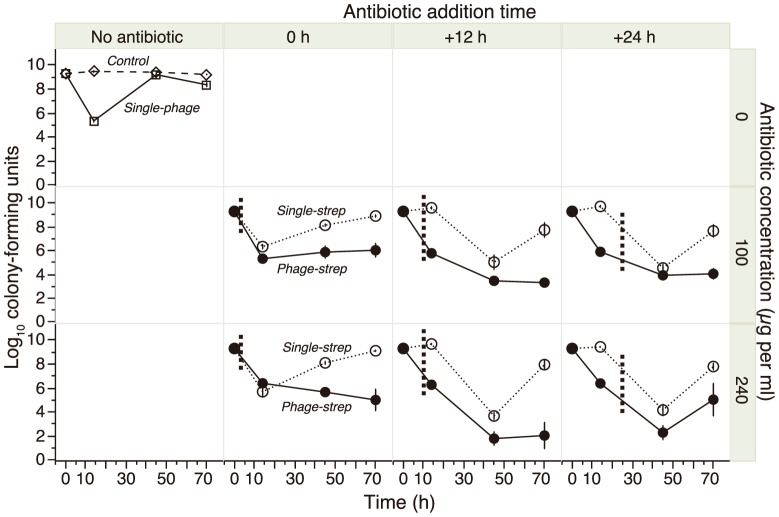
Changes in bacterial density over the course of the experiment. The six lower panels show the single antibiotic treatment (“single-strep”) and the combined phage-strep treatment, for different addition times (red dotted lines) of the antibiotic streptomycin and for two antibiotic doses. The top-left panel shows the single-phage treatment and the unexposed control lines.

**Figure 3:**
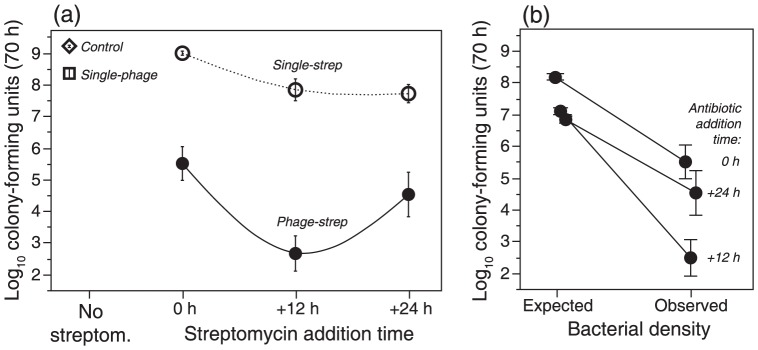
Final bacterial densities. (a) Effects of single treatments (single-strep, single-phage) and combined treatments (phage-strep), for different addition times of the antibiotic streptomycin in mean (± SE) final bacterial densities (70 h). Control lines were untreated, and ancestral bacteria regrown from frozen stocks for the assay. Note that the lines connect final densities for independently tested addition times, and do not represent time series of bacterial density. (b) Expected and observed density in the combined treatments, for the different antibiotic addition times. Expected density extrapolated from single treatments, assuming additive action of antibiotic and phage.

We challenged *P. aeruginosa* with either single or combined treatments of the phage LUZ7 and two doses of the antibiotic streptomycin (strep), administered at different time points. Bacterial population density was tracked over 70 h (Figure 1) to test the hypothesis that the use of phages can contribute to reduce antibiotic doses below the MIC, and that simultaneous or sequential administration of the two antimicrobials have different consequences on bacterial densities. Both single-phage and single-strep treatments strongly reduced bacterial density over the first 24 h, by up to 6 orders of magnitude (Figure 2). However, densities rebounded and nearly reached the levels of untreated controls by the end of the experiment (70 h) in all populations (Figure 2, 3A).

The combined phage-strep treatment caused a significantly stronger reduction in density compared to either single treatment (vs. single-phage: t_58_ = 3.60, p = 0.0007; vs. single-strep: t_105_ = 9.43, p<0.0001; Figure 3A). Unlike in the single treatments, almost 60% (30/54) of the populations did not recover from the combined treatment and showed strongly suppressed final densities (<10^5^ CFU/mL). We then evaluated the relative effects of simultaneous (0 h) or delayed (+12 h, +24 h) addition of strep to populations containing phage (Figure 1). We found that bacterial density reduction at the end of the experiment was maximal when the antibiotic was added with a +12 h delay (phage x strep addition time^2^ interaction: F_1, 95_ = 5.03, p = 0.0272). Streptomycin dose (100 vs. 240 µg/mL) had no significant effect on final density, nor were there significant interactions with other treatments (all p>0.25; Figure 2).

We further assessed whether the combined action of phage and antibiotic was additive or synergistic. To this end, we extrapolated outcomes in combined treatments from added effects on final bacterial density in the single treatments. Final densities were significantly lower than expected (F_1, 477_ = 278.0, p<0.0001; Figure 3B), indicating a positive synergistic action of phage and antibiotic. This positive synergy was most pronounced for the +12 h antibiotic addition time (expected/observed x strep addition time interaction: F_2, 477_ = 14.06, p<0.0001; Figure 3B). Thus, an intermediate time delay in antibiotic addition in the combined treatment resulted in the strongest negative impact on bacterial population density.

### 2. Antibiotic and phage resistance

**Figure 4:**
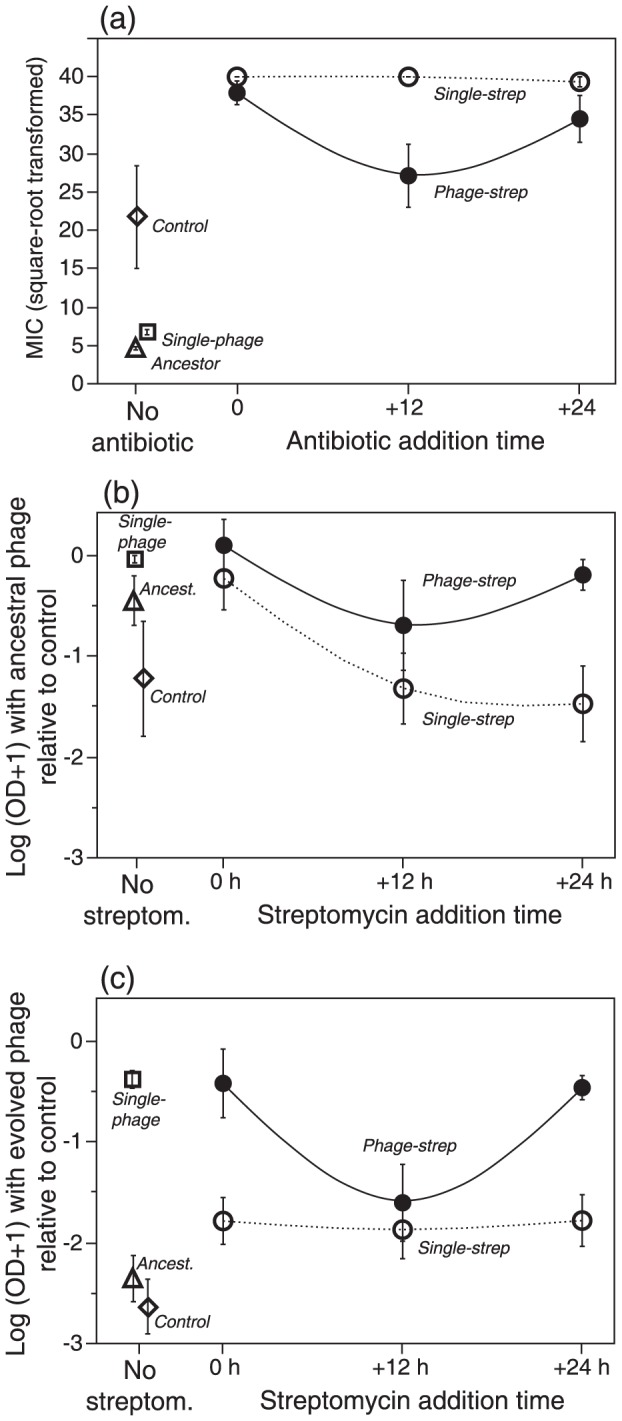
Mean (± SE) resistance of final bacterial populations (70 h) from single (strep or phage) and combined (phage-strep) treatments. Control lines were untreated, and ancestral bacteria regrown from frozen stocks for the assay (a) Streptomycin resistance, assessed as the Minimal Inhibitory Concentration (MIC, in µg/mL). (b) Resistance to ancestral phage, calculated as the difference in optical density (OD, log-transformed) (+1) of bacteria in the presence and absence of phage, measured in a 24-h growth assay. (c) Resistance to evolved phage, as measured in (b). Note that the lines connect final densities for independently tested addition times, and do not represent time series of bacterial density.

For the final bacterial populations (70 h) we analyzed variation in resistance to (ancestral) phage and to the antibiotic, the latter measured as the Minimum Inhibitory Concentration (MIC) of streptomycin. Bacteria from streptomycin treatments generally evolved very high levels of resistance (MIC≥800 µg/mL, Figure 4A). In the single-strep treatment, resistance reached maximum levels, whereas antibiotic resistance was lower in the combined phage-strep treatment (F_1, 79_ = 27.6, p<0.0001), but nonetheless higher than in the single-phage treatment (F_1, 40_ = 47.41, p<0.0001, Figure 4A). Resistance values were lower for populations where streptomycin was added to the phage with a +12 h delay (treatment x strep addition time^2^ interaction: F_1, 79_ = 4.48, p = 0.0375) compared to the other treatments. Note that while ancestral bacteria were fully susceptible to streptomycin, moderate increases in resistance were detected for bacteria from the single-phage treatment and for totally unexposed controls (Figure 4A).

As expected, bacteria from phage treatments were more resistant to phage than bacteria from no-phage treatments (F_2, 110_ = 5.90, p = 0.0037; Figure 4B). Specifically, the absence of phage tended to produce a decrease in resistance, suggesting a possible cost of resistance. Similar to the finding for antibiotic resistance, increases in phage resistance in the combined treatment tended to be minimal when strep was added with a +12 h delay (strep addition time: F_1, 32_ = 2.97, p = 0.0947). In the single-strep treatments, we observed a loss of phage resistance significantly affected by the antibiotic addition time (F_1, 45_ = 7.22, p = 0.01). Very similar results were obtained when resistance was measured against evolved (from 70 h) rather than ancestral phage, with a clear minimum when adding the antibiotic at +12 h (F_1, 32_ = 5.20, p = 0.0293, Figure 4C). Resistance against ancestral phage was generally higher than against evolved phage (across all replicates: t_33_ = 3.62, p = 0.001), suggesting adaptation of phage to contemporary bacteria.

Finally, we found no evidence for a trade-off between antibiotic resistance and phage resistance in the combined treatment. In fact, the across-population correlation between the two traits was positive rather than negative (r = 0.41, n = 34, p = 0.0154), indicating that higher levels of phage resistance were associated with higher antibiotic resistance.

## Discussion

There is increasing attention on alternative treatments against bacterial pathogens, due to the inevitability of antibiotic resistance and difficulties in developing new antibiotics [25]. The combination of antibiotics and phages for clinical or environmental applications is a tantalizing possibility, but it is not known whether phage therapy alone or in combination with antibiotics will improve on antibiotics alone in the short term, and reduce or prevent resistance in the long term. In this work, we study the combined effect of an antibiotic and phages on *Pseudomonas aeruginosa* bacterial population density and levels of evolved resistance, testing different application sequences and antibiotic doses. We show that combined treatments result in synergistic suppression of bacterial density and less resistance than either treatment alone, but also that the application sequence of both antimicrobials, and not antibiotic dose, is key to minimize the levels of resistance.

We found that combining phage and antibiotic results in lower bacterial density than expected from the addition of the respective single treatment effects (Figure 3). This positive synergism is consistent with previous observations in *P. fluorescens* [9] [26], and probably due to resistance mutation limitation as a result of lowered bacterial population size [27]. A possible explanation for this synergistic effect is a demographic feedback produced by the addition of phage [18], limiting the capacity of the bacteria to resist antibiotic exposure, as suggested by the detailed bacterial density dynamics (Figure 2). Bacteria were most affected when the antibiotic was applied when the phages themselves had their strongest impact on bacterial population density (+12 h addition time), suggesting an optimal window of opportunity in the implementation of combined therapies to restrain pathogens. For the other two application times, the synergistic effect was reduced. When the two agents are applied simultaneously, streptomycin is likely to constrain the efficacy of phages due to intensive host damage by means of protein synthesis inhibition [28]. If inhibition reduces the per-host cell output of phage, then overall phage titer may not be sufficient to cause massive reductions in bacterial cell density. Conversely, when the antibiotic is applied 24 hours after the phage, we argue that bacterial populations recover before being submitted to the antibiotic. This demographic feedback mechanism is consistent with ‘evolutionary rescue’, which links the demographic dynamics of population decline with the genetic dynamics of adaptation under rapid environmental deterioration [29]. Our results suggest that understanding the population dynamics and evolutionary biology of multiple interactive agents is important for the success of new therapies [18].

A possible evolutionary risk of antimicrobial compounds producing a synergistic effect in a combined treatment is that resistance mutations have a larger selective advantage compared to the single treatments, and as such the rate of adaptation will be higher [30] [31]. In our combined treatments, resistance to both phage and antibiotic increased relative to the ancestral bacteria, indicating positive responses to selection by both agents (as already shown separately for phages and antibiotics in *P. aeruginosa*; [32] [33], respectively). However, resistance levels did not exceed those of single treatments: they were equal or even lower (Figure 4). This suggests that our combined treatments did not lead to faster adaptation of the bacteria. Consistent with previous work [34], we observed an increase in antibiotic resistance in non-treated control populations relative to ancestral bacteria, possibly associated with biofilm formation, adaptation to the nutrient media or the emergence of low frequency antibiotic resistant mutants in the large populations occurring in our microcosms (>10^9^ cells/mL).

Interestingly, we show that an intermediate time delay between application of phage followed by an antibiotic leads to lower levels of bacterial resistance to either agent, as compared to shorter or longer delays, or to the application of either agent separately. Indeed, the sequential application of combined therapies has been suggested to generate lower levels of resistance compared to simultaneous addition, especially when the antimicrobial agents have different bacterial targets, as in a recent study employing phages that use different host receptors [35]. We also provide experimental support that in synergistic combinations it is possible to reduce antibiotic doses and still reduce bacterial populations significantly whilst limiting resistance [11]. Given the prediction that demographic and genetic changes interact, it is not unexpected that the effects of addition time on resistance mirrored those on bacterial density. The capacity of treated bacterial populations to recover and attain high densities is directly related to the increasing frequency of resistant mutants, and thus the higher mean population resistance levels observed at the end of our experiment. Nevertheless, in another study using distinct combinations of phages against bacteria, Hall and colleagues [35] argue that the effectiveness of multiphage therapy depends on the order and type of phages combined, indicating a more mechanistic constraint rather than the demographic one suggested here.

More generally, combined phage-antibiotic therapy may be expected to have an advantage over antibiotic cocktails of less cross-resistance because phage and antibiotics are fundamentally different regarding cellular mechanisms affected and the genetic changes resulting in resistance [18]. In particular, streptomycin resistance mutations typically involve the ribosomal protein S12 and 16S rRNA [36], whereas resistance mutations to a *Podovirus* such as LUZ7 usually require the alteration of lipopolysaccharide (LPS) components, the phage receptor on the bacterial outer membrane [37]. Here, we observed a general positive association between mean levels of phage and antibiotic resistance for the combined treatments, suggesting potentially unconstrained multiple resistance evolution, or in other words, that trade-offs between phage and antibiotic resistance do not appear to play a role [38]. It should be noted, however, that we measured resistance at the population level; a more precise analysis of the relationship would require measurements of individual bacterial genotypes to establish genetic correlations.

As an antimicrobial agent, bacteriophages are different from antibiotics in that the former can evolve or even coevolve with the bacteria, and therefore potentially limit resistance evolution during treatment [9] [18] [26]. How this (co)evolutionary component influences the efficiency and predictability of treatment outcomes is still largely unclear [23] [35] [39]. Here, we find evidence for the evolution of bacterial resistance to phage, but also evolutionary change in phage infectivity, in agreement with recent study [23]. Overall, bacterial resistance to phage from the end of the experiment was lower than that to the ancestral phage, clearly suggesting evolution of the phage towards increased infectivity. Levels of resistance to ancestral and evolved phage were highly correlated, indicating considerable coherence in treatment effects regarding resistance evolution. Future study needs to evaluate to what extent these patterns involve coevolutionary specificity, and what the implications are for longer-term pathogen control.

We acknowledge that *in vitro* studies such as ours will be limited in predicting outcomes in a clinical context, in which other important drivers of selection for pathogenic microbes include the host immune system and its spatial structure [40], and bacterial densities might be significantly lower [41]. In addition, we should expect that resistance to either phages and/or antibiotics will entail fitness costs for bacteria that could be accentuated *in vivo* [26]. Interestingly, phage resistance may lead to selection for less virulent bacterial variants, for example, through the loss of surface phage receptors that are also virulence determinants, as in the case of *Yersinia pestis* [42]. This possibility should be explored both *in vitro* and in hospital settings to evaluate if combined approaches at disinfection are also able to reduce the pathogenicity of bacteria surviving such treatments.

The use of combined antimicrobial therapies for the treatment of highly resistant pathogens has been applied in the clinic, for example, to combat *Mycobacterium tuberculosis*, HIV, and the malaria pathogen *Plasmodium falciparium* [43]. Even though it has achieved considerable success, the potential to reduce the rate of evolution of resistance to combination therapies need to reach more problematically resistant infectious diseases in the future [44]. A better understanding of the pharmacodynamics of combining phages and antibiotics will be vital to the eventual implementation of new therapeutic strategies targeting multiresistant nosocomial infections [19]. Our work shows that at an intermediate application time there is a window of opportunity, where mortality due to the antibiotic results in lower absolute populations and reduced resistance levels because bacterial populations are at low density, and both antibiotic and phage resistance mutations are less likely to be present [29].
